# 1-Cyano­methyl-4-aza-1-azoniabicyclo­[2.2.2]octane tetra­fluoro­borate monohydrate

**DOI:** 10.1107/S1600536810017757

**Published:** 2010-05-22

**Authors:** Ying Cai

**Affiliations:** a Ordered Matter Science Research Center, Southeast University, Nanjing 211189, People’s Republic of China

## Abstract

In the title compound, C_8_H_14_N_3_^+^·BF_4_^−^·H_2_O, the cation, anion and water molecule all lie on mirror planes. The BF_4_^−^ anion is disordered over two orientations with occupancies refined to 0.57 (2) and 0.43 (2). The water mol­ecule is linked to the cation *via* an O—H⋯N hydrogen bond. Weak inter­molecular O—H⋯F, C—H⋯O and C—H⋯F hydrogen bonds consolidate the crystal packing.

## Related literature

For applications of 1,4-diaza­bicyclo­[2.2.2]octane derivatives, see: Basaviah *et al.* (2003 ▶); Almarzoqi *et al.* (1986 ▶). For a related structure, see: Batsanov *et al.* (2005 ▶).
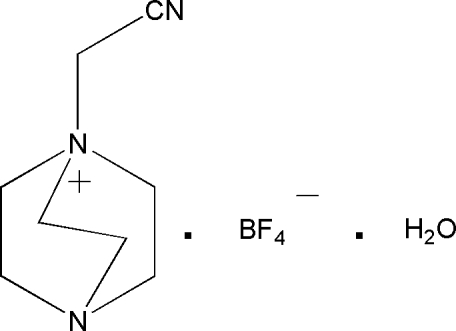


## Experimental

### 

#### Crystal data

C_8_H_14_N_3_^+^·BF_4_^−^·H_2_O
*M* *_r_* = 257.05
Orthorhombic, 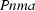

*a* = 17.288 (4) Å
*b* = 6.8663 (14) Å
*c* = 9.776 (2) Å
*V* = 1160.5 (4) Å^3^
*Z* = 4
Mo *K*α radiation
μ = 0.14 mm^−1^
*T* = 293 K
0.20 × 0.20 × 0.20 mm

#### Data collection

Rigaku Mercury CCD diffractometer
Absorption correction: multi-scan (*CrystalClear*; Rigaku, 2005 ▶) *T*_min_ = 0.691, *T*_max_ = 1.000
10255 measured reflections
1239 independent reflections
1043 reflections with *I* > 2σ(*I*)
*R*_int_ = 0.037

#### Refinement

*R*[*F* ^2^ > 2σ(*F* ^2^)] = 0.057
*wR*(*F* ^2^) = 0.140
*S* = 1.03
1239 reflections
115 parameters
28 restraints
H-atom parameters constrained
Δρ_max_ = 0.78 e Å^−3^
Δρ_min_ = −0.43 e Å^−3^

### 

Data collection: *CrystalClear* (Rigaku, 2005 ▶); cell refinement: *CrystalClear*; data reduction: *CrystalClear*; program(s) used to solve structure: *SHELXS97* (Sheldrick, 2008 ▶); program(s) used to refine structure: *SHELXL97* (Sheldrick, 2008 ▶); molecular graphics: *SHELXTL* (Sheldrick, 2008 ▶); software used to prepare material for publication: *SHELXL97*.

## Supplementary Material

Crystal structure: contains datablocks I, global. DOI: 10.1107/S1600536810017757/cv2716sup1.cif

Structure factors: contains datablocks I. DOI: 10.1107/S1600536810017757/cv2716Isup2.hkl

Additional supplementary materials:  crystallographic information; 3D view; checkCIF report

## Figures and Tables

**Table 1:** Hydrogen-bond geometry (Å, °)

*D*—H⋯*A*	*D*—H	H⋯*A*	*D*⋯*A*	*D*—H⋯*A*
O1—H1*WB*⋯N1^i^	0.85	2.06	2.903 (3)	175
O1—H1*WA*⋯F1^ii^	0.85	2.53	3.29 (2)	150
O1—H1*WA*⋯F1^iii^	0.85	2.53	3.29 (2)	150
C3—H3*B*⋯O1^iv^	0.96	2.58	3.474 (3)	155
C5—H5*A*⋯F1^v^	0.96	2.32	3.140 (7)	143
C5—H5*A*⋯F1^vi^	0.96	2.54	3.231 (8)	129

Symmetry codes: (i) 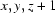; (ii) 
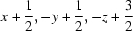
; (iii) 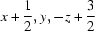; (iv) 
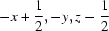
; (v) 
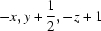
; (vi) 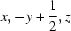.

**Fig. 1.**
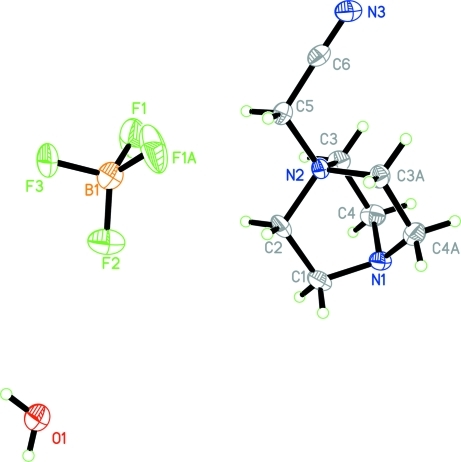
A view of the title compound with the atomic numbering scheme [symmetry code: (A) x, -y+1/2, z]. Displacement ellipsoids were drawn at the 30% probability level. Only major parts of disordered F atoms are shown.

## supplementary crystallographic information

### Comment

1,4-Diazabicyclo[2.2.2]octane (DABCO) is used as an excellent organocatalyst for a variety of reactions because of the nucleophilicity (Basaviah *et al.*, 2003), which can even go through substitution with relatively unreactive electrophiles such as dichloromethane (Almarzoqi *et al.*, 1986). We report here the crystal structure of the title compound, [C_8_H_14_N_3_]^+^.BF_4_^-^.H_2_O (I), which was obtained by the solution evaporation method.

The reaction of Bromoacetonitrile and DABCO proceeds quickly in CH_3_CN, leading to the immediate precipitation of 1-(cyanomethyl)-4-aza-1-azonia-bicyclo[2.2.2]octane bromide.

The stucture of the title compound, (I), is shown in Fig. 1. All bond lengths and angles in (I) are normal and comparable with those observed in the related compound (Batsanov *et al.*, 2005). In the crystal struture of the title compound, all moieties are situated on mirror planes and the F atoms of the BF_4_^-^ anion are disordered. Lattice water molecule is paired with the cation by O—H···N hydrogen bond (Table 1). The crystal packing is stabilized by weak intermolecular hydrogen bonds of C—H···O, C—H···F and O—H···F (Table 1).

### Experimental

Bromoacetonitrile (0.1 mol, 12.00 g) and 1,4-diaza-bicyclo[2.2.2]octane (0.05 mol, 5.6 g) were dissolved in CH_3_CN(40 ml) with stirring for 1 hour at room temperature. The white product formed was 1-(cyanomethyl)-4-aza-1-azonia-bicyclo[2.2.2]octane bromide which was filtered, washed with acetonitrile and dried with 80% yield. A mixture of 1-(cyanomethyl)-4-aza-1-azonia-bicyclo[2.2.2]octane bromide (0.01 mol 2.32 g) and tetrafluoro-borate sodium (0.01 mol 1.10 g) in H_2_O (20 ml) was stirred until clear. After slow evaporation, colourless plate crystals of the title compand suitable for X-ray analysis were obtained with about 60% yield.

The powder-pressed pellets of compound 1 were used in temperature-dependent dielectric measurements because of the difficulty in obtaining large crystals. There is no dielectric anomaly observed between 93 K and 353 K. So there may no structural phase transitions between this temperature range.

### Refinement

C-bound H atoms were geometrically positioned with C—H = 0.96 Å. O-bound H atoms were located in a difference Fourier map, and then placed in idealized positions with O—H = 0.85 Å. All H atoms were refined as riding, with U_iso_(H) = 1.2-1.5 U_eq_(C, O).

### Crystal data

**Table:**

C_8_H_14_N_3_^+^·BF_4_^−^·H_2_O	*F*(000) = 536
*M**_r_* = 257.05	*D*_x_ = 1.471 Mg m^−^^3^
Orthorhombic, *P**n**m**a*	Mo *K*α radiation, λ = 0.71073 Å
Hall symbol: -P 2ac 2n	Cell parameters from 3350 reflections
*a* = 17.288 (4) Å	θ = 6.3–55.3°
*b* = 6.8663 (14) Å	µ = 0.14 mm^−^^1^
*c* = 9.776 (2) Å	*T* = 293 K
*V* = 1160.5 (4) Å^3^	Prism, colourless
*Z* = 4	0.20 × 0.20 × 0.20 mm

### Data collection

**Table:**

Rigaku Mercury CCD diffractometer	1239 independent reflections
Radiation source: fine-focus sealed tube	1043 reflections with *I* > 2σ(*I*)
graphite	*R*_int_ = 0.037
Detector resolution: 13.6620 pixels mm^-1^	θ_max_ = 26.0°, θ_min_ = 3.2°
ω scans	*h* = −21→21
Absorption correction: multi-scan (*CrystalClear*; Rigaku, 2005)	*k* = −8→8
*T*_min_ = 0.691, *T*_max_ = 1.000	*l* = −12→12
10255 measured reflections	

### Refinement

**Table:**

Refinement on *F*^2^	Secondary atom site location: difference Fourier map
Least-squares matrix: full	Hydrogen site location: inferred from neighbouring sites
*R*[*F*^2^ > 2σ(*F*^2^)] = 0.057	H-atom parameters constrained
*wR*(*F*^2^) = 0.140	*w* = 1/[σ^2^(*F*_o_^2^) + (0.053*P*)^2^ + 1.0547*P*] where *P* = (*F*_o_^2^ + 2*F*_c_^2^)/3
*S* = 1.03	(Δ/σ)_max_ < 0.001
1239 reflections	Δρ_max_ = 0.78 e Å^−^^3^
115 parameters	Δρ_min_ = −0.43 e Å^−^^3^
28 restraints	Extinction correction: *SHELXL97* (Sheldrick, 2008)
Primary atom site location: structure-invariant direct methods	Extinction coefficient: 0.0476 (15)

### Special details

**Table:**

Geometry. All esds (except the esd in the dihedral angle between two l.s. planes) are estimated using the full covariance matrix. The cell esds are taken into account individually in the estimation of esds in distances, angles and torsion angles; correlations between esds in cell parameters are only used when they are defined by crystal symmetry. An approximate (isotropic) treatment of cell esds is used for estimating esds involving l.s. planes.
Refinement. Refinement of *F*^2^ against ALL reflections. The weighted *R*-factor wR and goodness of fit *S* are based on *F*^2^, conventional *R*-factors *R* are based on *F*, with *F* set to zero for negative *F*^2^. The threshold expression of *F*^2^ > σ(*F*^2^) is used only for calculating *R*-factors(gt) etc. and is not relevant to the choice of reflections for refinement. *R*-factors based on *F*^2^ are statistically about twice as large as those based on *F*, and *R*- factors based on ALL data will be even larger.

### Fractional atomic coordinates and isotropic or equivalent isotropic displacement parameters (Å^2^)

**Table:**

	*x*	*y*	*z*	*U*_iso_*/*U*_eq_	Occ. (<1)
N2	0.10929 (12)	0.2500	0.2012 (2)	0.0298 (5)	
N3	−0.08170 (15)	0.2500	0.1105 (3)	0.0501 (7)	
C6	−0.03141 (17)	0.2500	0.1853 (3)	0.0397 (7)	
C3	0.11555 (11)	0.0716 (3)	0.1121 (2)	0.0396 (5)	
H3A	0.0741	0.0701	0.0467	0.048*	
H3B	0.1119	−0.0437	0.1671	0.048*	
C2	0.17505 (17)	0.2500	0.3022 (3)	0.0445 (8)	
H2A	0.1721	0.1366	0.3595	0.053*	
C5	0.03436 (16)	0.2500	0.2790 (3)	0.0414 (7)	
H5A	0.0320	0.3632	0.3365	0.050*	
N1	0.23841 (13)	0.2500	0.0745 (3)	0.0407 (6)	
C1	0.25193 (18)	0.2500	0.2228 (3)	0.0507 (9)	
H1A	0.2815	0.1369	0.2471	0.061*	
C4	0.19356 (12)	0.0767 (4)	0.0382 (2)	0.0471 (6)	
H4A	0.1850	0.0765	−0.0588	0.057*	
H4B	0.2226	−0.0379	0.0609	0.057*	
O1	0.36760 (14)	0.2500	0.8848 (2)	0.0574 (6)	
H1WB	0.3318	0.2500	0.9440	0.086*	
H1WA	0.4139	0.2500	0.9130	0.086*	
B1	0.0837 (3)	0.2500	0.6715 (5)	0.0574 (6)	
F1	0.0498 (10)	0.113 (2)	0.5939 (6)	0.108 (4)	0.57 (2)
F2	0.1630 (5)	0.2500	0.6946 (10)	0.076 (3)	0.57 (2)
F3	0.0555 (16)	0.2500	0.800 (2)	0.083 (5)	0.57 (2)
F1'	0.0855 (8)	0.0727 (12)	0.6066 (13)	0.083 (3)	0.43 (2)
F2'	0.1513 (11)	0.162 (3)	0.6819 (16)	0.086 (3)	0.216 (12)
F3'	0.039 (2)	0.2500	0.792 (3)	0.080 (5)	0.43 (2)

### Atomic displacement parameters (Å^2^)

**Table:**

	*U*^11^	*U*^22^	*U*^33^	*U*^12^	*U*^13^	*U*^23^
N2	0.0319 (12)	0.0335 (12)	0.0240 (11)	0.000	−0.0017 (9)	0.000
N3	0.0342 (13)	0.0579 (18)	0.0581 (17)	0.000	0.0006 (13)	0.000
C6	0.0341 (15)	0.0424 (16)	0.0426 (17)	0.000	0.0113 (13)	0.000
C3	0.0406 (11)	0.0365 (11)	0.0417 (11)	−0.0033 (9)	0.0008 (9)	−0.0105 (9)
C2	0.0448 (17)	0.060 (2)	0.0291 (14)	0.000	−0.0143 (13)	0.000
C5	0.0384 (16)	0.0548 (19)	0.0310 (15)	0.000	0.0066 (12)	0.000
N1	0.0290 (12)	0.0525 (15)	0.0405 (14)	0.000	−0.0018 (10)	0.000
C1	0.0355 (16)	0.072 (2)	0.0443 (18)	0.000	−0.0126 (14)	0.000
C4	0.0410 (11)	0.0499 (13)	0.0505 (12)	0.0043 (10)	0.0030 (10)	−0.0130 (11)
O1	0.0541 (12)	0.0670 (14)	0.0512 (12)	0.000	0.0061 (10)	0.000
B1	0.0541 (12)	0.0670 (14)	0.0512 (12)	0.000	0.0061 (10)	0.000
F1	0.149 (7)	0.125 (6)	0.051 (2)	−0.086 (5)	−0.010 (3)	−0.012 (3)
F2	0.050 (3)	0.074 (8)	0.105 (4)	0.000	−0.012 (3)	0.000
F3	0.081 (13)	0.135 (6)	0.031 (4)	0.000	0.006 (5)	0.000
F1'	0.097 (5)	0.056 (3)	0.096 (5)	−0.003 (3)	0.014 (4)	−0.026 (3)
F2'	0.065 (6)	0.068 (8)	0.124 (7)	0.036 (5)	−0.003 (5)	−0.013 (6)
F3'	0.058 (9)	0.116 (8)	0.064 (9)	0.000	0.018 (6)	0.000

### Geometric parameters (Å, °)

**Table:**

N2—C5	1.502 (3)	C1—H1A	0.9599
N2—C2	1.506 (3)	C4—H4A	0.9600
N2—C3	1.507 (2)	C4—H4B	0.9600
N2—C3^i^	1.507 (2)	O1—H1WB	0.85
N3—C6	1.136 (4)	O1—H1WA	0.85
C6—C5	1.460 (4)	B1—F2'^i^	1.319 (16)
C3—C4	1.530 (3)	B1—F2'	1.319 (16)
C3—H3A	0.9600	B1—F3	1.34 (2)
C3—H3B	0.9600	B1—F1	1.345 (7)
C2—C1	1.540 (4)	B1—F1^i^	1.345 (7)
C2—H2A	0.9600	B1—F1'	1.373 (9)
C5—H5A	0.9600	B1—F1'^i^	1.373 (9)
N1—C4	1.464 (3)	B1—F2	1.391 (9)
N1—C4^i^	1.464 (3)	B1—F3'	1.41 (3)
N1—C1	1.468 (4)		
			
C5—N2—C2	108.6 (2)	H4A—C4—H4B	108.0
C5—N2—C3	110.78 (13)	H1WB—O1—H1WA	117.9
C2—N2—C3	108.96 (14)	F2'^i^—B1—F3	104.4 (13)
C5—N2—C3^i^	110.78 (13)	F2'—B1—F3	104.4 (13)
C2—N2—C3^i^	108.96 (14)	F2'^i^—B1—F1	138.6 (9)
C3—N2—C3^i^	108.7 (2)	F2'—B1—F1	96.2 (17)
N3—C6—C5	178.8 (3)	F3—B1—F1	111.6 (8)
N2—C3—C4	108.53 (17)	F2'^i^—B1—F1^i^	96.2 (17)
N2—C3—H3A	109.9	F2'—B1—F1^i^	138.6 (9)
C4—C3—H3A	110.1	F3—B1—F1^i^	111.6 (8)
N2—C3—H3B	110.0	F2'^i^—B1—F1'	114.9 (8)
C4—C3—H3B	109.9	F3—B1—F1'	116.0 (5)
H3A—C3—H3B	108.4	F1^i^—B1—F1'	111.8 (10)
N2—C2—C1	108.7 (2)	F2'—B1—F1'^i^	114.9 (8)
N2—C2—H2A	110.0	F3—B1—F1'^i^	116.0 (5)
C1—C2—H2A	109.9	F1—B1—F1'^i^	111.8 (10)
C6—C5—N2	110.7 (2)	F1'—B1—F1'^i^	124.9 (10)
C6—C5—H5A	109.6	F3—B1—F2	101.9 (13)
N2—C5—H5A	109.4	F1—B1—F2	121.4 (10)
C4—N1—C4^i^	108.7 (2)	F1^i^—B1—F2	121.4 (10)
C4—N1—C1	108.90 (16)	F1'—B1—F2	93.0 (7)
C4^i^—N1—C1	108.90 (16)	F1'^i^—B1—F2	93.0 (7)
N1—C1—C2	111.1 (2)	F2'^i^—B1—F3'	115.1 (14)
N1—C1—H1A	109.2	F2'—B1—F3'	115.1 (14)
C2—C1—H1A	109.6	F1—B1—F3'	103.3 (11)
N1—C4—C3	111.76 (18)	F1^i^—B1—F3'	103.3 (11)
N1—C4—H4A	108.8	F1'—B1—F3'	113.5 (6)
C3—C4—H4A	109.3	F1'^i^—B1—F3'	113.5 (6)
N1—C4—H4B	109.4	F2—B1—F3'	114.1 (15)
C3—C4—H4B	109.5		

Symmetry codes: (i) *x*, −*y*+1/2, *z*.

### Hydrogen-bond geometry (Å, °)

**Table:**

*D*—H···*A*	*D*—H	H···*A*	*D*···*A*	*D*—H···*A*
O1—H1WB···N1^ii^	0.85	2.06	2.903 (3)	175
O1—H1WA···F1^iii^	0.85	2.53	3.29 (2)	150
O1—H1WA···F1^iv^	0.85	2.53	3.29 (2)	150
C3—H3B···O1^v^	0.96	2.58	3.474 (3)	155
C5—H5A···F1^vi^	0.96	2.32	3.140 (7)	143
C5—H5A···F1^i^	0.96	2.54	3.231 (8)	129

Symmetry codes: (ii) *x*, *y*, *z*+1; (iii) *x*+1/2, −*y*+1/2, −*z*+3/2; (iv) *x*+1/2, *y*, −*z*+3/2; (v) −*x*+1/2, −*y*, *z*−1/2; (vi) −*x*, *y*+1/2, −*z*+1; (i) *x*, −*y*+1/2, *z*.
